# Feline Cryptococcosis: Two Case Reports and a Literature Review

**DOI:** 10.3390/pathogens15030279

**Published:** 2026-03-04

**Authors:** Stanisław Dzimira

**Affiliations:** Department of Pathology, Faculty of Veterinary Medicine, Wrocław University of Environmental and Life Sciences, Norwid Str. 31, 50-375 Wroclaw, Poland; stanislaw.dzimira@upwr.edu.pl

**Keywords:** cryptococcosis, cytology, fine-needle aspiration, feline fungal infections, One Health

## Abstract

Cryptococcosis is a severe systemic mycosis affecting humans and animals, caused primarily by members of the *Cryptococcus neoformans*/*Cryptococcus gattii* species complex. In cats, it is the most common systemic fungal infection and may present with non-specific signs involving the upper respiratory tract, skin, lymph nodes, eyes, or the central nervous system. This study presents two feline cases of cryptococcosis diagnosed by cytological examination and provides an updated literature review. Fine-needle aspiration biopsies were performed in two cats with chronic nasal swelling and submandibular enlargement. Cytological smears stained with hematoxylin and eosin revealed spherical to oval yeast-like organisms with a characteristic thick, non-staining capsule, narrow-based budding, and absence of pseudohyphae, consistent with *Cryptococcus* spp. Based on cytological findings, both patients were treated with oral itraconazole, resulting in favorable clinical outcomes. A limitation of this study is the lack of mycological culture or molecular confirmation, owing to the owners’ refusal of further diagnostic testing. These cases highlight the diagnostic value of cytology as a rapid tool for differentiating fungal infections from neoplastic processes. Early diagnosis and antifungal therapy are crucial for successful management. From a One Health perspective, feline cryptococcosis may indicate shared environmental exposure risks relevant to both animal and human health.

## 1. Introduction

Cryptococcosis is a severe systemic mycosis affecting humans and animals, caused predominantly by members of the *Cryptococcus neoformans*/*Cryptococcus gattii* species complex (CNGSC). In humans, the disease is primarily observed in immunocompromised individuals, including patients with HIV (Human Immunodeficiency Virus) infection, lymphatic neoplasia, organ transplantation, or corticosteroid therapy, and remains associated with significant mortality [1,2,3].

In veterinary medicine, cryptococcosis is most commonly diagnosed in cats and represents the most frequent systemic fungal infection in this species. Unlike in humans, infection in cats may occur in both immunocompromised and apparently immunocompetent individuals. Clinical presentation typically involves the nasal cavity, skin, lymph nodes, eyes, and central nervous system [4,5,6,7,8,9,10,11,12,13,14].

*C. neoformans* and *C. gattii* are environmental pathogens whose primary reservoirs include soil and decomposing organic matter. Birds, particularly pigeons, may contribute to environmental contamination. Infection most commonly occurs through inhalation of aerosolized propagules (basidiospores or desiccated yeast cells). Although other pathogenic species such as *C. laurenti* have been reported and may exhibit reduced antifungal susceptibility, CNGSC remains the principal cause of disease in companion animals [2,3].

Cases of cryptococcosis in animals have been reported in North and South America, Europe, Asia, and Australia, highlighting its global distribution [4,5,6,7,8,9,10,11,12,13,14]. In human medicine, epidemiological studies of SARS-CoV-2 infections indicate that respiratory failure and decreased immunity observed in patients with COVID-19 increase the risk of opportunistic fungal infections, including cryptococcosis [15,16].

Despite increasing recognition of feline cryptococcosis, diagnostic and therapeutic challenges remain in routine veterinary practice.

The present study describes two cases of feline cryptococcosis diagnosed by cytological examination and provides an updated review of recent literature.

## 2. Materials and Methods

### Cases Presentation

**Figure 1:**
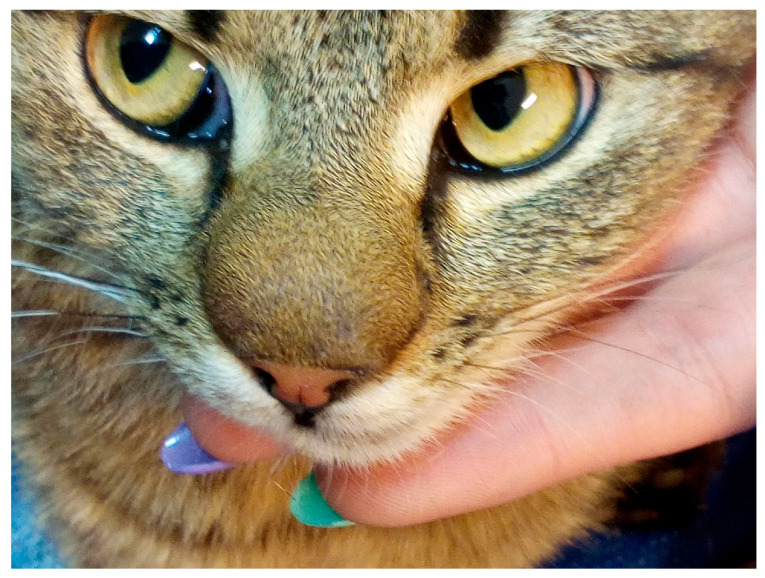
Patient no. 1, enlarged outline of the nose without superficial skin damage.

The examination material consisted of cytological preparations from cats. The first of them was a free-living, rarely house-dwelling European cat, a neutered male aged approximately 9 years. In the cat, a thickening approximately 1.5 cm in diameter appeared on the nose and persisted for quite a long time (approximately 3 months), which worried the caretaker. The cat showed no other signs of disease and no interest in the lesion that had appeared. The lesion was quite soft in consistency and painless (Figure 1).

The second patient was a rarely outdoor-going domestic cat, a male aged 1 year and 2 months, in which enlargement of the left submandibular region and swallowing difficulties were observed. Retroviral status (FIV/FeLV) was not available for either cat.

From both patients, material was collected by fine-needle aspiration biopsy using an injection needle (21 G) and a 10 mL syringe, in order to exclude neoplastic proliferations. A smear of the collected material on microscope slides was stained with hematoxylin and eosin (HE) using a routine laboratory protocol. The preparations were viewed under an Olympus BX53 light microscope (Olympus, Tokyo, Japan) coupled to an Olympus UC90 camera. Measurements were performed using cellSens Standard V.1 software.

## 3. Results

### 3.1. Cytological Findings

**Figure 2:**
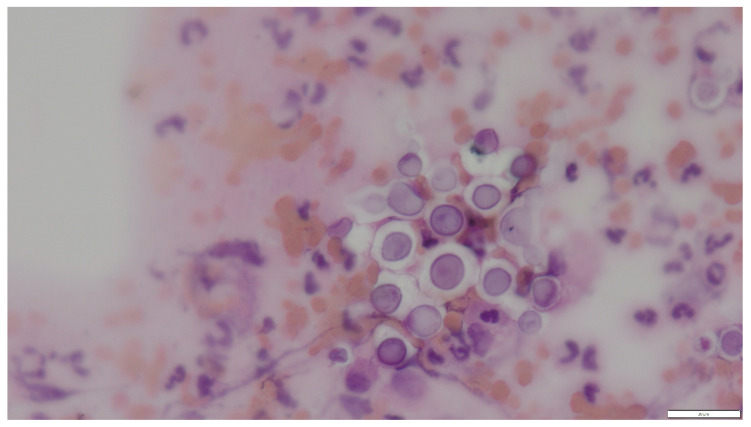
Case 1. Numerous round and oval yeast-like cells with a characteristic, distinct, non-staining capsule. Hematoxylin and eosin staining, magnification 600×, bar 20 µm.

**Figure 3:**
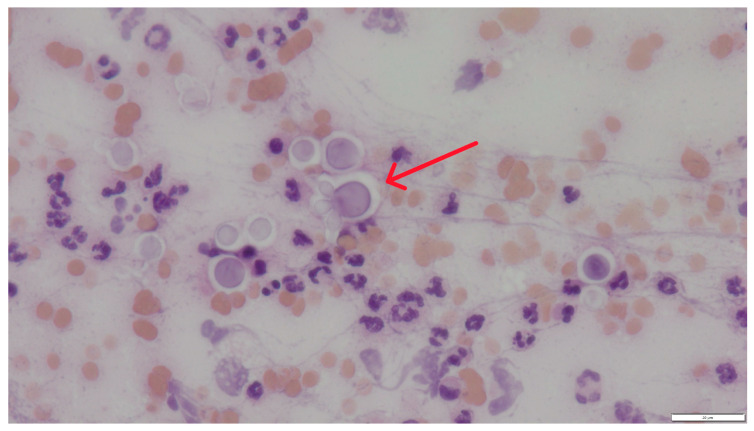
Case 2. Fungal cells forming narrow-based budding (red arrow). Hematoxylin and eosin staining, magnification 600×, bar 20 µm.

**Figure 4:**
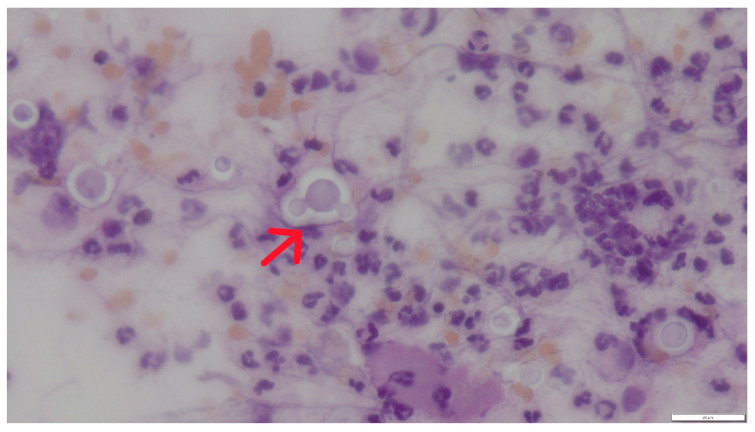
Case 1. Cytological preparation illustrating narrow-based budding (red arrow) and mixed inflammatory infiltrate composed predominantly of neutrophils. Hematoxylin and eosin staining, magnification 600×, bar 20 µm.

In cytological preparations, numerous spherical to oval yeast-like cells with a characteristic, non-staining capsule were observed, occurring singly or in loose aggregates. Their diameter was usually approximately 10 (without capsule) to 16 (with capsule) µm (Figure 2). The cells showed characteristic narrow-based budding, being one of the key features differentiating *Cryptococcus* spp. from other yeasts (Figure 3 and Figure 4). No formation of pseudohyphae or true hyphae was observed, which additionally argues against infections caused by *Candida* spp. or mould fungi.

Cytological examination revealed numerous spherical yeast organisms approximately 10 µm in diameter, characterized by a distinct, thick, non-staining capsule visible as a bright “halo” surrounding the cells. Narrow-based budding was observed, and pseudohyphae were absent. The background was highly cellular, with abundant neutrophils and lymphocytes, indicating an active inflammatory response. These features are characteristic of *Cryptococcus* spp., particularly members of the *C. neoformans*/*C. gattii* species complex (CNGSC) [7,10,11,12,13,14].

Based on these findings, a diagnosis of highly probable *Cryptococcus* spp. infection was established.

### 3.2. Treatment and Outcome

Given the owners’ unwillingness to undergo further, more detailed examinations, and based on the cytological results, itraconazole (Itracovet 10 mg/mL, AB7 Santé) was prescribed orally at 5 mg/kg body weight once daily for 6 weeks. Liver enzyme activity was monitored during treatment and remained within reference ranges. Clinical improvement was observed within the first 2–3 weeks, and complete resolution of lesions was achieved after 6 weeks. No adverse effects were reported. Long-term follow-up beyond treatment completion was not available.

## 4. Discussion

Fungi are ubiquitous organisms that can cause mycoses in humans and animals. The role of animals in the epidemiology of human mycoses is diverse. They can be treated as vectors of human mycoses, e.g., dermatophytozoonoses caused by *Microsporum canis*, common in cats and dogs. According to Tampieri, the pattern of human dermatophilic mycoses has changed in Italy over the last century: at the beginning of the century anthropophilic fungi dominated, whereas currently zoophilic fungi are the main cause [17].

Another aspect raised by Tampieri is animal substrate as a growth factor for pathogenic fungi: “animalization” of soil (i.e., adding such remnants as hair, skin scales, droppings, and other organic substances) creates an optimal substrate for the growth and multiplication of geophilic or saprophytic fungi, such as *Microsporum gypseum* and *Cryptococcus neoformans*. The current human lifestyle, often in close contact with bird populations, wild animals, domestic mammals, and anthropogenic animals, as well as with human-populated areas, appears to favor the development of environments conducive to the proliferation of certain pathogenic fungi, thereby leading to infections in humans and animals [17].

Cryptococcosis is the most common systemic fungal disease in cats worldwide; it is caused by the *Cryptococcus neoformans*–*Cryptococcus gattii* species complex, which comprises eight genotypes and several subtypes (strains) with varied geographic distributions, pathogenicities, and susceptibilities to antimicrobial agents. Infection most commonly occurs through inhalation of aerosolized environmental propagules (desiccated yeast cells or basidiospores) originating from contaminated soil, organic debris, or avian excreta, leading initially to colonization of the nasal cavity and upper respiratory tract. The prognosis is favorable in most cases, provided that diagnosis and treatment are initiated sufficiently early. Fungal infections caused by pathogenic fungi from the *C. neoformans*/*gattii* species complex (CNGSC) have been described in companion animals, mainly cats and dogs [4,10,13,18,19,20,21,22,23].

In cats, cryptococcosis most commonly develops following inhalation of infectious propagules, with primary colonization of the nasal cavity and paranasal sinuses. From this site, local tissue invasion may occur, with possible extension to the central nervous system through the cribriform plate. Hematogenous dissemination may subsequently involve multiple organs, including the skin and CNS [14]. Cutaneous lesions in cats are therefore most often considered a manifestation of disseminated disease rather than a primary site of infection. However, primary cutaneous involvement has been occasionally suggested.

Unlike in dogs, immunosuppression appears to play a less consistent role in feline cryptococcosis. Several reports describe infection in apparently immunocompetent cats, supporting the hypothesis that *Cryptococcus neoformans* may act as a primary pathogen in this species [8,11]. Nevertheless, concurrent retroviral infections such as FIV may impair antifungal immunity and predispose to systemic disease in some cases [7].

Cooley et al., using computed tomography (CT), examined 9 cats and 12 dogs and showed that imaging features of nasal cryptococcosis are limited. This retrospective, single-center, consecutive case series aimed to describe CT features of lesions in cats and dogs with nasal cryptococcosis. Images were assessed by a veterinary radiologist for the presence of a nasal or nasopharyngeal mass, regional destruction, intracranial extension, and lymphadenopathy. Most cats (8/10 [80%]) had lesions in the nose, with most cases described as non-destructive/non-invasive rhinitis (7/8 [88%]). None of the cats had cribriform plate destruction or meningeal enhancement. All dogs had nasal lesions. Eleven dogs (11/12 [92%]) had rhinitis with turbinate lysis. Most dogs (8/12; 67%) had a nasal mass, and in seven dogs the caudal nasal mass extended into the rostral portion of the nasopharynx. Most dogs had cribriform plate destruction (9/12 [75%]). The results confirm potential differences in CT appearance between dogs and cats with nasal cryptococcosis [21].

Wronski et al. conducted a retrospective study of 428 cats that died from various diseases. Histopathological ocular lesions were identified in 29% of cases (approximately 124/428 cats). Among these cats with ocular lesions, 41% were attributed to infectious causes, including viral infections (FIV, FIP) and *Cryptococcus* spp. [23]. The most commonly reported ocular abnormalities associated with infectious etiologies were anterior or posterior uveitis, panuveitis, optic neuritis, and meningitis. These findings indicate that ocular involvement may occur secondary to systemic infectious diseases, including cryptococcosis. However, such lesions are not always clinically apparent, as microscopic changes may be present without obvious macroscopic abnormalities.

Fungal infections causing inflammatory response, proliferative changes, and deformation of tissues in the infected area require inclusion in the differential diagnosis of neoplastic lesions. Cytological examination of biopsies and swabs, or histopathological examination of tissue sections, is a useful method for differentiating these lesions [12,23,24,25]. An advantage of securing material for histopathological examination is the possibility of performing additional tests, such as immunohistochemical assays. Choi et al. described a case of lymphadenopathy clinically indistinguishable from feline lymphoma due to *C. neoformans* infection, underscoring the need to include cryptococcosis in the differential diagnosis of cats with lymphadenopathy. Cytological examination of biopsies is the first diagnostic method for differentiating disease lesions [22].

However, it is important to note that a negative cytology result does not completely exclude the diagnosis of cryptococcosis, particularly in cases where the fungal presence is minimal or the sampled lesion is not representative. The most reliable diagnostic method for fungal infections is PCR, which Pennisi et al. recommend for the diagnosis and therapeutic management of infections caused by CNGSC fungi in cats [26]. Studies conducted by a group of Japanese researchers included multilocus sequence typing analysis and antifungal drug susceptibility testing of 14 *Cryptococcus* spp. strains originating from domestic cats in Japan and one strain isolated from a cat in Singapore. In susceptibility testing of these strains to antifungal drugs, one strain exceeded the epidemiological cutoff value (ECV) for amphotericin B and 5-fluorocytosine, and two strains exceeded the ECV for fluconazole. The authors stated that examination of properties, including resistance, of *Cryptococcus* spp. strains carried by cats living in close proximity to humans may contribute to improved health in both cat populations and humans [9]. Similar conclusions can be drawn from the results of Florek et al. 2021, who demonstrated, in about 10% of the population examined by them, *C. neoformans* strains isolated from the environment, MIC values against 5-fluorocytosine exceeding 64 mg/L, which indicates the presence of resistant strains in the environment [27].

Trivedi et al. analyzed cases of cryptococcosis in cats and dogs in California and demonstrated differences in species distribution and tissue involvement compared with previously reported data from Australia [5]. In their study, *C. gattii* was more commonly identified in cats, whereas *C. neoformans* predominated in dogs. The authors suggested that strain variation and geographic factors may influence host preference and lesion distribution. These findings indicate that clinical presentation may vary depending on the infecting species and molecular type, highlighting the importance of regional epidemiology and comprehensive diagnostic approaches beyond antigen testing alone.

Evans et al. described a case of *Cryptococcus albidus* infection in a 6-year-old spayed female cat with acute respiratory signs and pleural effusion, confirmed by molecular studies. The initial diagnosis was based on cytological examination of the effusion, and mycological and biochemical tests identified the yeast as *C. albidus,* susceptible to all tested antifungal drugs. However, the subsequent 18S polymerase chain reaction showed 99% homology with the *Cryptococcus neoformans* strain and only 92% homology with the *C. albidus* strain. Atypical cytological morphology in this case emphasizes the need for additional studies to identify the fungus. Although *C. albidus* should be considered a potential feline pathogen, when encountering such rare species other than *C. neoformans*, PCR confirmation of their presence is recommended [20]. Myers et al. diagnosed and treated cases of atypical cutaneous infections of cutaneous cryptococcosis. Histological examination of sections collected from lesions showed severe granulomatous or pyogranulomatous and eosinophilic dermatitis with rare yeasts lacking a capsule. Immunohistochemistry, PCR, and fungal culture confirmed that the etiological agent in these cases was Cryptococcus spp [6]. Huang 2023 described a case of cryptococcosis of the central nervous system caused by *C. neoformans* in a 3-year-old female cat [28].

A somewhat similar case to ours was described by McEwan et al.; in a 6-year-old, spayed domestic female cat, kept exclusively indoors, noisy breathing was reported in the history. Skull radiographs revealed increased soft tissue density in the caudal portion of the left nasal cavity. Computed tomography and rhinoscopy revealed a mass in the caudal naris and a smaller lesion almost completely blocking airflow through the nasal passages. Using rhinoscopy, a sample was collected from a fleshy, yellow-brown mass visible in the caudal nasopharynx. Histopathological examination confirmed *Cryptococcus* spp. Systemic antifungal treatment with fluconazole were implemented [29].

Toth et al. described a 13-year-old European shorthair cat in which progressive skin, nasal-ocular lesions, and weight loss had been present for 3 months. Cytology of a skin scraping showed the presence of numerous macrophages with numerous extracellular and intracytoplasmic microorganisms consistent with the genus *Cryptococcus.* Histopathological examination showed inflammation of the pyogranuloma type with yeasts surrounded by a capsule. The result of the latex agglutination test for cryptococcal antigen in serum was positive. In fungal culture, *Cryptococcus neoformans* was detected [14].

Cytological examination is widely used as an initial diagnostic method in cases of suspected cryptococcosis and, in many reports, has provided sufficient evidence to initiate treatment. The identification of encapsulated yeasts with narrow-based budding is highly characteristic of *Cryptococcus* spp. and allows a rapid presumptive diagnosis in clinical practice. Although fungal culture and molecular methods remain the gold standard for species-level identification, they are not always readily available in routine veterinary settings. Molecular characterization and antifungal susceptibility testing of feline *Cryptococcus* isolates have demonstrated geographic variability and the presence of strains with reduced susceptibility to selected antifungal agents [9,27,28]. These findings highlight the epidemiological importance of species- and genotype-level identification, particularly in regions where cats live in close contact with humans.

In the present cases, diagnosis was based solely on cytological examination of fine-needle aspirates. The lack of culture or molecular confirmation represents a limitation, as species-level identification was not achieved. However, the clear cytological features combined with the favorable clinical response to antifungal therapy support the practical diagnostic value of cytology in similar clinical circumstances.

Differential diagnoses should also include other systemic mycoses, depending on geographic location (e.g., histoplasmosis or blastomycosis), atypical encapsulated yeasts, and infectious or non-infectious granulomatous conditions, including sterile pyogranulomatous processes. Careful cytological assessment combined with clinical context remains essential for narrowing the diagnostic spectrum [30,31].

Recent reports of feline cryptococcosis and diagnostic approaches are summarized in Table 1.

## 5. Conclusions

The presented cases highlight the diagnostic utility of cytological examination in cats with proliferative or inflammatory lesions of the nasal region and lymph nodes. In both patients, characteristic cytomorphological features allowed prompt initiation of antifungal therapy, resulting in favorable clinical outcomes. Although species-level confirmation was not performed, the therapeutic response supports the reliability of cytological diagnosis in selected clinical contexts. Feline cryptococcosis should be considered in the differential diagnosis of chronic nasal and subcutaneous lesions, particularly when encapsulated yeasts are observed. Further studies incorporating molecular identification are needed to better characterize the epidemiology of *Cryptococcus* infections in Poland.

An integrated diagnostic approach combining cytology or histopathology with cryptococcal antigen detection and, when available, mycological culture provides reliable confirmation of infection in feline patients, as reported in previous studies [7,8,10,11,12,13,14]. Although culture-based and molecular methods may require longer turnaround times, they remain important for species- and genotype-level identification, particularly for epidemiological and research purposes.

Long-term azole therapy remains the cornerstone of treatment for feline cryptococcosis. Treatment duration depends on clinical response and laboratory monitoring. Serial measurement of cryptococcal antigen titers has been reported to be useful for assessing therapeutic response and detecting relapse, especially in cases involving the central nervous system or disseminated disease [7,10,11,12,14,20,22,25,26,29,30].

From a broader perspective, feline cryptococcosis should be considered within the One Health framework, given the shared environmental reservoirs of *Cryptococcus* spp. and their capacity to cause severe disease in both animals and humans. Cats may serve as sentinel species, signaling environmental exposure risks and highlighting the importance of interdisciplinary collaboration among veterinarians, microbiologists, and medical professionals.

## Figures and Tables

**Table 1:** Selected reports of feline cryptococcosis published between 2013 and 2025.

Ref.	Author (Year)	Country	Cat (Age/Sex)	Main Clinical Signs	Diagnostic Methods	Species/Type	Treatment
[6]	Myers et al. (2017)	USA	Four cats, 6–13 y, 2 NM,2 FS	Skin lesions	Histopathology, histochemistry, IHC, culture, PCR, and sequencing	*Cryptococcus* spp.	NR
[7]	de Fátima Lazameth- Diniz et al. (2025)	Brazil	20 mo, M	Systemic disease + FIV + demodicosis	Cytology, culture, PCR-RFLP	*C. gattii* VGII	Fluconazole
[8]	Teh et al. (2024)	Australia	Ragdoll, 13 y, FS	Unilateral (progressing to bilateral) ocular discharge, sneezing, nasal discharge, weight loss	CrAg assay, LCAAT, histopathology	*C. neoformans complex*	euthanised
[9]	Omura et al. (2024)	Japan, Singapore	Domestic cats (n = 14)	Respiratory/systemic signs	Culture, MLST, susceptibility testing	*Cryptococcus neoformans*, *C. gattii*	NR
[10]	Costa et al. (2022)	Brazil	Siamese, 6 y, M	Lymphadenomegaly only	Cytology, culture, sequencing	*Cryptococcus* sp.	Fluconazole
[11]	Olivares et al. (2021)	Costa Rica	1,5 y, NM	Subcutaneous lesion/tumor in the neck	Cytology, culture, MALDI TOF	*C. neoformans (var. grubii)*	Fluconazole
[12]	Rösch et al. (2024)	Germany	2 y, Norwegian Forest, M	Nasopharyngeal mass mimicking neoplasia	Imaging, cytology, histopathology, PCR	*Cryptococcus neoformans var. grubii.*	Itraconazole
[13]	Glavinić et al. (2024)	Bosnia & Herzegovina	2 y, F	wound	Cytology, histopathology, LCAAT	*C. neoformans*	Itraconazole
[14]	Tóth et al. (2025)	Hungary	13 y, FS	Nasal–ocular lesions, weight loss	Cytology, histopathology, antigen test, culture, MALDI TOF	*C. neoformans*	Itraconazole
[20]	Evans et al. (2018)	USA	6 y, FS	Acute respiratory signs, pleural effusion, cranial mediastinal mass	Cytology, culture, PCR	*C. neoformans*/ *C. albidus*	Fluconazole
[22]	Choi et al. (2025)	Korea	Persian cat, 7 y, NM	Systemic lymphadenopathy mimicking lymphoma	Cytology, culture	*C. neoformans*	Itraconazole, Fluconazole
[25]	Oronan et al. (2013)	Philippines	4 y, M	Sneezing and nasal, ocular and gingival discharges	Cytology	*Cryptococcus* spp.	Fluconazole
[29]	McEwan et al. (2022)	USA	6 y, FS	Nasopharyngeal mass, noisy breathing	Histopathology, CrAg assay	*Cryptococcus* spp.	Fluconazole
—	Present study (2025)	Poland	9 y, MN/1 y, M	Nasal swelling/submandibular enlargement	Cytology	*Cryptococcus* spp.	Itraconazole

## Data Availability

Data are contained within the article.

## Abbreviations

The following abbreviations are used in this manuscript:

CNGSC	*C. neoformans*/*C. gattii* species complex
HIV	Human Immunodeficiency Virus
SARS-CoV-2	Severe Acute Respiratory Syndrome Coronavirus 2
COVID-19	Coronavirus disease 2019
HE	Hematoxylin and eosin
CNS	Central Nervous System
FeLV	Feline Leukemia Virus
PCR-RFLP	Polymerase Chain Reaction Restriction Fragment Length Polymorphism
ECV	Epidemiological Cutoff Value
CT	Computed Tomography
FIV	Feline Immunodeficiency Virus
FIP	Feline Infectious Peritonitis
MIC	Minimum Inhibitory Concentration
NM	Neutered Male
M	Male
F	Female
FS	Female Spayed
MALDI TOF	Matrix-Assisted Laser Desorption/Ionization—Time of Flight
LCAAT	Latex Cryptococcus Antigen Agglutination Test
MLST	Multilocus Sequence Typing
CrAg	Cryptococcal Antigen
